# Depressive, anxiety, and post-traumatic stress symptoms affecting hospitalized and home-isolated COVID-19 patients: a comparative cross-sectional study

**DOI:** 10.1186/s43045-021-00105-9

**Published:** 2021-05-04

**Authors:** Amany Elshabrawy Mohamed, Amira Mohamed Yousef

**Affiliations:** Psychiatry Department, Faculty of Medicine, Zagazig University, Zagazig, Egypt

**Keywords:** COVID-19, Psychiatric symptoms, Isolation

## Abstract

**Background:**

Coronavirus has affected more than 100 million people. Most of these patients are hospitalized in isolation wards or self-quarantined at home. A significant percentage of COVID-19 patients may experience psychiatric symptoms. This study attempts to assess depressive, anxiety, and post-traumatic stress symptoms in home-isolated and hospitalized COVID-19 patients, besides whether the isolation setting affected these symptoms’ presentation.

**Results:**

The study involved 89 patients with confirmed COVID-19 virus, and the patients were divided into 2 groups: 43 patients in the home-isolated group (group A) and 46 patients in the hospital-isolated group (group B).

The majority of subjects were male and married; also, they were highly educated. 30.2% from group A and 47.8% from group B had a medical occupation. There was a statistically significant difference (*p*= 0.03) between both groups in the presence of chronic disease. There was a statistically significant increase in suicidal thoughts in the home-isolated group (37.2%) (*p* = 0.008**). We found a statistically significant increase in the abnormal scores of Hospital Anxiety Depression Scale–Depression (HADS–Depression) in the home-isolated group (69.7%) compared to the hospital-isolated group (32.6%) (*p* <0.001**) which denotes considerable symptoms of depression. Moreover, we found that (32.6%) from the home-isolated group and (39.1%) from the hospital-isolated group had abnormal scores of Hospital Anxiety Depression Scale–Anxiety (HADS–Anxiety) which denotes considerable symptoms of anxiety. Also, we found 66.7% and 87.2% scored positive by the Davidson Trauma Scale (DTS) in the home-isolated group and hospital-isolated group, respectively. Which was statistically significant (*p* = 0.02**). On doing a binary logistic regression analysis of HADS and DTS with significantly related independent factors, we revealed that lower education levels and family history of psychiatric disorder were risk factors for abnormal HADS–Anxiety scores in COVID-19 patients. The medical occupation was a protective factor against having abnormal HADS–Depression scores in COVID-19 patients, while home isolation was a risk factor. On the contrary, the medical occupation was a risk factor for scoring positive in DTS in COVID-19 patients. Simultaneously, low levels of education and home isolation were protective factors.

**Conclusion:**

A significant number of patients diagnosed with the COVID-19 virus develop depressive, anxiety, and post-traumatic stress symptoms, whether they were isolated in the hospital or at home; besides, the isolation setting may affect the presenting symptoms.

## Background

Diseases triggered by viral infections are predominant, and some are known to cause neuropsychiatric disorders involving cognitive, affective, behavioral, and perceptual symptoms [1].

Scientists found multiple cases of atypical pneumonia in Wuhan, China, in December 2019, and WHO later discovered a virus called SARS-coronavirus 2. (SARS-CoV-2) had inflicted these effects [2].

More than 50 million victims of COVID-19 have been affected. Many of these patients are hospitalized in advanced and segregated hospitals or isolated in their residences. Those afflicted with a novel and potentially lethal disease feel terrified, rage, and anxious for friends’ and families’ health [3].

Early literature investigated the respiratory manifestations of COVID-19 patients; nevertheless, evolving data indicate that neuropsychiatric illnesses have been elevated in a significant number of patients, particularly those with severe infection [4, 5]. A joint case series documenting neuropsychiatric symptoms in 153 patients suggested that a compromised mental state was the most predominant presentation in the sample population. However, this study did not investigate the initial psychiatric manifestations, such as depression and anxiety [6, 7].

Generally, there has been milieu research concerning the epidemiological impact on the populace, not in those subjected to SARS-2-CoV directly [7, 8]. However, the literature on COVID-19 patients indicates elevated rates of anxiety as well as depression in this group [8].

Hospitalized COVID-19 patients could be demographically at higher risk of experiencing psychiatric symptoms due to their serious illness, the stress of staying in isolation whilst hospitalized, in addition to the potential side effects of drugs as well as treatments. On the contrary, patients isolated at home may also be prone to developing specific psychiatric symptoms, which may be illustrated through the dissemination dynamics of COVID-19 that parallel with dehumanizing reports of widely used social networks that led to increased self-perceived post-traumatic stress, suicidality, and other psychological symptoms [7, 9]. In addition to being alone, even at home, with negative emotions such as functional disability, stigma, anxiety, phobia, irritation, and resentment may be a trigger factor for developing psychiatric symptoms [9].

COVID-19 is marked by its expansion beyond the officially affiliated health organizations. The requisite restricting procedures resulted in a daunting situation where anxiety and confusion prevailed [10], in addition to the apprehension of mortality upon infection, stigmatization, and prejudice for COVID-19 patients. In addition to fear of infecting others may induce various psychiatric symptoms [11]. These symptoms may increase when isolated at home and with close contact with their relatives and not under direct and immediate medical treatment.

Environmental pressure in lockdown, difficulty breathing, and other types of complaints in patients during the COVID-19 disease outbreak caused a wide range of psychiatric disorders similar to SARS [12]. These circumstances caused the secretion of stressor-dependent corticotrophin-releasing hormone (CRH) from hypothalamic paraventricular nucleus (PVN) neurons as well, which binds to its receptors in the anterior pituitary gland causing stimulation of the hypothalamic-pituitary-adrenal (HPA) axis) [12], besides causing elevated ACTH levels, which in turn leads to elevated concentrations of glucocorticoids [13]. When a high concentration of glucocorticoids is released and binds to glucocorticoid receptors (GR) in the brain, hyper-activation of the HPA axis causes stress-related gene expression [14]. Hyper-activation of the HPA axis and dysfunction in the stress-related gene lead to increased exposure to environmental stress [15]. Environmental stress can alter the epigenetics of SERT, BDNF, GR, FKBP5, and CRHR1 genes via multiple mechanisms and induces transcriptional changes of these gene expressions, resulting in stress-related disorders [16]. Thus, the extreme stressful environment caused by COVID-19 can lead to an increased psychiatric disorder.

Moreover, SARS-CoV-2 can also affect brain tissue by causing a cytokine storm, which is believed to affect psychiatric symptoms [17]. The researchers assumed that the low-grade inflammatory response might be crucial to the psychiatric symptoms of COVID-19 infection [18]. To date, it cannot be determined whether or not the virus has an independent effect on inflammation and mental health [19].

We aimed to assess depressive, anxiety, and post-traumatic stress symptoms in home-isolated and hospitalized COVID-19 patients, besides whether the isolation setting affected these symptoms’ presentation.

## Methods

### Study design and setting

The current study is a comparative cross-sectional study on the Sharkia Governorate, where patients were recruited from Zagazig University Hospitals’ isolation hospital. The isolated home patients were residents of Sharkia Governorate, Egypt, between 22 May 22 and 28 July 2020.

### Study participants

Eighty-nine subjects were recruited in this study and were divided into two groups: 43 home-isolated patients (group A) and 46 hospital-isolated patients (group B).

Patients were qualified to participate in this study, depending on the requirements defined by the study protocol.

The inclusion criteria:

1. Confirmed mild and moderate cases of COVID-19 infection (patients with oxygen saturation > 92%; respiratory rate ≤ 30 breaths/min; lung infiltrates < 50%).
2. Age ≥ 18 years old
3. Both genders were included.
4. All socioeconomic classes were included.

A verified case of COVID is determined by positive RT-PCR examination of samples from pharyngeal and nasal cavities.

Exclusion criteria:

1. Presence of mental retardation, dementia, or delirium.
2. Refusal to sign the consent.
3. Confirmed severe cases of COVID-19 infection (patients with oxygen saturation < 92%; respiratory rate ≥ 30 breaths/min; lung infiltrates > 50%).

### Sampling

Since the research is novel and no published articles were used for sample size calculation, we carried out a pilot study. The pilot study was a crucial stage in the research, and we conducted it to estimate the expected difference between home and hospital isolation. Besides, it identifies potential problems and deficiencies in the research tools and protocol before implementation during the whole study [20].

The pilot study was conducted using a small sample of 20 patients (10 home isolated and 10 hospitals isolated). This pilot study revealed that the DTS score in home isolation versus hospital isolation was 22.2 ± 8.0 vs 27.1 ± 8.2 at a confidence level of 95% and 80% power. This finding was used to calculate the research sample size, which was 89 COVID-19 patients. The sample size was calculated by the OPEN EPI software package [21].

Patients in the pilot study were excluded from the original research sample due to the tool modifications and avoid inter-observer bias.

### Tools and operational procedures

All participants enrolled in this study were subjected to the following (through an online Google form and phone call):

1. The online Google form that was sent to the patients to respond consisted of four sections:
  *The first section* explained the current study’s aim and procedures. It included an obligatory inquiry with a yes or no answer representing the participants’ acceptance or refusal to participate in our research.
  *The second section* included the semi-structured COVID-19 questionnaire, which contained obligatory questions to collect sociodemographic data (age, sex, marital status, education. occupation, and residence) and clinical data (having a chronic disease, smoking, personal, or family history of the psychiatric disorder), and questions that assisted some psychiatric symptoms of the subjects (mood status, having obsessive or paranoid thoughts, hallucinations, memory, and concentration).
  *The third and fourth section* included the patients’ psychometric assessments via the Arabic version of the Hospital Anxiety and Depression Scale (HADS) and the Arabic version of the Davidson Trauma Scale (DTS).

### The Arabic version of (HADS)

This scale is used for screening depression and anxiety. It includes 14 questions: 7 questions for the anxiety component (HADS−Anxiety) and 7 questions for the depression component (HADS–Depression). The scores for anxiety and depression ranged from 0 to 21 points. Levels 1–7 were considered normal, whereas 8–10 is regarded as borderline, > 11 considered abnormal and denoted considerable symptoms of anxiety or depression. The internal consistency of the scales was 87% and 81%, respectively [22]. Terkawi AS and his collaborators performed a systematic translation process to translate the original English HADS into Arabic. They documented that the Cronbach α for the HADS–Anxiety subscale was 0.83 (95% confidence interval 0.79–0.88) and for the HADS–Depression subscale was 0.77 (0.7–0.83). HADS–Anxiety score was strongly correlated with GAD-7, and HADS–Depression score was strongly associated with MDI. Besides, the results showed adequate internal consistency of HADS subscales among the patients [23]. The HADS questionnaire has been validated in many languages, countries, and settings, including general practice and community settings. HADS has been used in the HUNT study in Norway, a large community setting study done on the general population [24]. In our research, the scale showed excellent internal consistency with a Cronbach alpha coefficient of 0.84.

### The Arabic version of the Davidson Trauma Scale (DTS)

This scale is used for diagnosing post-traumatic stress disorder (PTSD). The DTS is a 17-item questionnaire that tests for PTSD symptoms. Objects are rated on a five-point scale (0 = “not at all distressing” to 4 = “extremely distressing”) to measure the degree of distress they produce. Participants are asked to define the most impactful event that has influenced them and how much trouble they are experiencing. Cut off point of 40 was used to distinguish between positive and negative cases. The test-retest reliability was *r* = 0.86 and internal consistency was *r* = 0.99 [25, 26]. It was translated to Arabic by Abdul Aziz Thabet, and Sady and Badr validated it in their study with a sample of adolescent sons of martyrs in Jablah, Syria. Cronbach alpha coefficient was 0.762 [27]. In our study, the scale showed excellent internal consistency with a Cronbach alpha coefficient of 0.8.

The HADS and DTS scales were tested for content validity by two panels of the Psychiatric Department experts. These experts assessed the tools for clarity, relevance, comprehensiveness, applicability, and understanding.

The reliability of the HADS and DTS scales was tested by measuring their internal consistency. It demonstrated an excellent level of reliability (Cronbach’s alpha = 0.84 and 0.81), respectively.

### Statistical analysis

The statistical analysis was performed using SPSS software version 27 [28], and then the data were presented in tables. Quantitative data were introduced as mean, standard deviation, and range, whereas qualitative data were presented as frequencies and proportions. Kolmogorov-Smirnov and Levene tests were conducted to evaluate the distribution characteristics of variables and homogeneity of variance.

Pearson’s chi-squared test (*χ*^2)^, Fisher’s exact test, and chi-square for linear trend were used to analyze qualitative variables as appropriate. Mann-Whitney (MW) *U* test was conducted to analyze quantitative data. Binary logistic regression analysis was performed to remove confounding factors. The *P* value of ˂ 0.05 was considered statistically significant.

## Results

The study involved 89 patients with confirmed COVID-19 virus, and the patients were divided into two groups: 43 patients in the home-isolated group (group A) and 46 patients in the hospital-isolated group (group B).

### Results of the sociodemographic data

The majority of the participants in both groups were male (67.4%) from group A and (69.6%) from group B and were highly educated.

The mean age was 39.9 ± 8.8 in group A and 41.3 ± 9.3 in group B.

(72.1%) from group A and 60.9% from group B were married.

(30.2%) from group A and 47.8% from group B had a medical occupation (Table 1).

**Table 1:** Comparison between home-isolated patients and hospital-isolated patients in demographic, clinical characteristics, and psychiatric symptoms

Variables		Home isolation (***n*** = 43) Group A	Hospital isolation (***n*** = 46) Group B	***p***
**Demographic characteristics**	Age (years):			
	Mean ± SD	39.9 ± 8.8	41.3 ± 9.3	0.5
	Sex, *n* (%):			
	Male	29 (67.4%)	32 (69.6%)	0.8
	Female	14 (32.6%)	14 (30.4%)	
	Marital status, *n* (%):			
	Married	31 (72.1%)	28 (60.9%)	0.4
	Single	5 (11.6%)	7 (15.2%)	
	Divorced	5 (11.6%)	4 (8.7%)	
	Widow	2 (4.7%)	7 (15.2%)	
	Education, *n* (%):			
	Primary and preparatory education	4 (9.3%)	2 (4.3%)	0.8
	Secondary education	6 (14.0%)	5 (10.9%)	
	High education	23 (53.4%)	28 (60.9%)	
	Post-graduate education	10 (23.3%)	11 (23.9%)	
	Occupation, *n* (%):			
	Medical	13 (30.2%)	22 (47.8%)	0.09
	Non-medical	30 (69.8%)	24 (52.2%)	
	Residence, *n* (%):			
	Urban	19 (44.2%)	26 (56.5%)	0.2
	Rural	24 (55.8%)	20 (43.5%)	
**Clinical characteristics**	Chronic disease, *n* (%):	12 (27.9%)	23 (50.0%)	0.03**
	Smoking, *n* (%):	10 (23.3%)	17 (37.0%)	0.2
	History of psychiatric disorder, *n* (%):			
	Depression	5 (11.6%)	3 (6.5%)	0.5
	Insomnia	2 (4.7%)	1 (2.2%)	
	Anxiety	3 (7.0%)	1 (2.2%)	
	OCD	0 (0.0%)	1 (2.2%)	
	Family history of psychiatric disorder, *n* (%):			
	Depression	5 (11.6%)	7 (15.2%)	0.7
	OCD	3 (7.0%)	4 (8.7%)	
	Insomnia	3 (7.0%)	1 (2.2%)	
	Bipolar disorder	2 (4.7%)	2 (4.3%)	
**Psychiatric symptoms**	Mood status:			
	Positive (relaxed, calm, cheerful)	5 (15.6%)	11 (23.9%)	0.4
	Negative (nervous, irritated, sad)	27 (84.4%)	35 (76.1%)	
	Loss of concentration	42 (97.7%)	40 (87.0%)	0.1
	Weakness of memory	39 (90.7%)	37 (80.4%)	0.2
	Annoying, urgent, and repetitive thoughts	17 (39.5%)	11 (23.9%)	0.1
	Paranoid thoughts	5 (11.6%)	9 (19.6%)	0.3
	Suicide thoughts	16 (37.2%)	6 (13.0%)	0.008**
	Hallucinations	0 (0.0%)	3 (6.5%)	0.2

Test of significance: Pearson’s chi-squared test, Fisher’s exact test, and Student’s *t* test

**Statistical significance

### Results of the health-related conditions

Ten patients from the home-isolated group and 6 from the hospital-isolated group had a history of psychiatric disorder.

We found that 27.9% from the home-isolated group and 50% from the hospital-isolated group had a chronic disease which was a statistically significant difference *p*= 0.03.

There was a statistically significant increase in the suicidal thoughts in the home-isolated group (37.2%) compared to hospital-isolated group (13%) (*p* = 0.008**). (Table 1).

### Hospital anxiety and depression scale results

HADS scores were calculated for all patients. The results were categorized according to the scale into three categories: normal, borderline, and abnormal Scores for depression and anxiety. The abnormal scores denote considerable symptoms of anxiety or depression (Table 2).

**Table 2:** Comparison between home-isolated patients and hospital-isolated patients in HADS and DTS and dual diagnosis

	Variables	Home isolation (***n*** = 43) Group A	Hospital isolation (***n*** = 46) Group B	***p***
**HADS and DTS**	HADS–Anxiety:			
	Mean ± SD	8.6 ± 2.4	9.3 ± 2.2	0.1
	• Normal (0–7)	19 (44.2%)	17 (37.0%)	
	• Borderline (8–10)	10 (23.3%)	11 (23.9%)	0.3
	• Abnormal (11–21)	14 (32.6%)	18 (39.1%)	
	HADS-Depression:			
	Mean ± SD	12.2 ± 3.6	9.8 ± 2.7	˂ 0.001**
	• Normal (0–7)	6 (14.0%)	13 (28.3%)	
	• Borderline (8–10)	7 (16.3%)	18 (39.1%)	0.003**
	• Abnormal (11–21)	30 (69.7%)	15 (32.6%)	
	Davidson Trauma Scale:			
	Mean ± SD	22.8 ± 4.3	28.5 ± 5.9	˂ 0.001**
	• Positive DTS (≥ 35)	28 (66.7%)	41 (87.2%)	
	• Negative DTS (˂ 35)	14 (33.3%)	6 (12.8%)	0.02**
**Having both abnormal scores in HADS and positive results in DTS**	Abnormal scores in HADS–Anxiety and positive results in DTS:			
	Present	5 (11.6%)	14 (30.4%)	0.03**
	Absent	38 (88.4%)	32 (69.6%)	
	Abnormal scores in HADS–Depression and positive results in DTS:			
	Present	21 (48.8%)	11 (23.9%)	0.01**
	Absent	22 (51.2%)	35 (76.1%)	
	Abnormal scores in both HADS–Depression and HADS–Anxiety:			
	Present	9 (20.9%)	4 (8.7%)	0.1
	Absent	34 (79.1%)	42 (91.3%)	

Test of significance: Student’s *t* test and chi-square for linear trend

**Statistical significance

We found a statistically significant increase in the abnormal scores of HADS–Depression in the home-isolated group (69.7%) compared to the hospital-isolated group (32.6%) (*p* < 0.001**) (Table 2).

Our results showed that 32.6% from the home-isolated group and 39.1% from the hospital-isolated group had abnormal scores of HADS–Anxiety which was statistically non-significant (Table 2).

### Davidson Trauma Scale results

DTS scores were calculated for all patients. The results were categorized according to the scale into two categories: positive and negative (Table 2).

We found that 66.7% and 87.2% scored positive by the Davidson Trauma Scale (DTS) in the home-isolated group and hospital-isolated group, respectively, which was statistically significant (*p* = 0.02**). There was a statistically significant increase (*p* ˂ 0.001**) in DTS scores among the hospital-isolated group with a mean score of 28.5 ± 5.9 compared to the home-isolated group with a mean score of 22.8 ± 4.3 (Table 2).

### Results regarding having both abnormal scores in HADS and positive results in DTS

We found that 30.4% in the hospital-isolated group and 11.6% in the home-isolated group had both abnormal scores in HADS–Anxiety and positive results in DTS, which was statistically significant (*p*= 0.03 **). On the contrary, we found that 48.8% in the home-isolated group and 23.9% in the hospital-isolated group had both abnormal scores in HADS–Depression and positive results in DTS, which was statistically significant (*p*= 0.01 **) (Table 2).

### Results regarding the association between HADS–Anxiety and demographic and clinical characteristics of the studied patients

Our result showed a statistically significant association between having abnormal HADS–Anxiety scores and [the female sex (*p* **<** 0.001**) lower education levels (*p*= 0.02**), non-medical occupation (*p* **<** 0.001**), and the presence of a family history of psychiatric disorder (*p*=0.002**)] (Table 3).

**Table 3:** Association between HADS–Anxiety and demographic and clinical characteristics of the studied patients

Demographic and clinical characteristics	Normal		Borderline		Abnormal		***P***
	No.	%	No.	%	No.	%	
Age:							
˂ 40 years old (*n* = 44)	23	52.3	8	18.2	13	29.5	0.07
≥ 40 years old (*n* = 45)	13	28.9	13	28.9	19	42.2	
Sex:							
Male (*n* = 61)	35	57.4	7	11.5	19	31.1	<0.001**
Female (*n* = 28)	1	3.6	14	50.0	13	46.4	
Marital status:							
Married (*n* = 59)	28	47.5	10	16.9	21	35.6	0.06
Single/divorced/widow (*n* = 30)	8	62.5	11	0.0	11	37.5	
Education level:							
Primary, preparatory, and secondary education (*n* = 17)	4	23.5	2	11.8	11	64.7	0.02**
High- and post-graduate education (*n* = 72)	32	44.4	19	26.4	21	29.2	
Occupation:							
Medical (*n* = 35)	23	65.7	3	8.6	9	25.7	<0.001**
Non-medical (*n* = 54)	13	24.1	18	33.3	23	41.6	
Residence:							
Urban (*n* = 45)	20	44.4	7	15.6	18	40.0	0.2
Rural (*n* = 44)	16	36.4	14	31.8	14	31.8	
Chronic disease (*n* = 35)	14	40.0	5	14.3	16	45.7	0.2
Smoking (*n* = 27)	14	51.9	3	11.1	10	37.0	0.1
History of psychiatric disorder (*n* = 16)	5	31.3	2	12.5	9	56.2	0.2
Family history of psychiatric disorder (*n* = 27):	6	22.2	4	14.8	17	63.0	0.002**

Test of significance: chi-square for linear trend

**Statistical significance

### Results regarding the association between HADS–Depression and demographic and clinical characteristics of the studied patients

Our results showed a statistically significant association between having abnormal HADS–Depression scores and [the female sex (*p*=0.01**) non-medical occupation (*p* **<** 0.001**), and the presence of a family history of psychiatric disorder (*p*=0.003**)] (Table 4).

**Table 4:** Association between HADS–Depression and demographic and clinical characteristics of the studied patients

Demographic and clinical characteristics	Normal		Borderline		Abnormal		***P***
	No.	%	No.	%	No.	%	
Age:							
˂ 40 years old (*n* = 44)	13	29.5	11	25.0	20	45.5	0.1
≥ 40 years old (*n* = 45)	6	13.3	14	31.1	25	55.6	
Sex:							
Male (*n* = 61)	18	29.5	14	23.0	29	47.5	0.01**
Female (*n* = 28)	1	3.6	11	39.3	16	57.1	
Marital status:							
Married (*n* = 59)	17	28.8	15	25.4	27	45.8	0.06
Single/divorced/widow (*n* = 30)	2	6.7	10	33.3	18	60.0	
Education level:							
Primary, preparatory, and secondary education (*n* = 17)	4	23.5	2	11.8	11	64.7	0.2
High- and post-graduate education (*n* = 72)	15	20.8	23	31.9	34	47.2	
Occupation:							
Medical (*n* = 35)	14	40.0	7	20.0	14	40.0	<0.001**
Non-medical (*n* = 54)	5	9.3	18	33.3	31	60.9	
Residence:							
Urban (*n* = 45)	13	28.9	11	24.4	21	46.7	0.2
Rural (*n* = 44)	6	13.6	14	31.8	24	54.5	
Chronic disease (*n* = 35)	12	34.2	8	22.9	15	42.9	0.6
Smoking (*n* = 27)	9	33.3	7	25.9	11	40.7	0.1
History of psychiatric disorder (*n* = 16)	5	31.3	5	31.3	6	37.4	0.4
Family history of psychiatric disorder (*n* = 27)	8	29.6	1	3.7	18	66.7	0.003**

Test of significance: chi-square for linear trend

**Statistical significance

### Results regarding the association between the Davidson Trauma Scale and demographic and clinical characteristics of the studied patients

Our results revealed a statistically significant association between positive DTS scores and (the female sex (*p*= 0.01**), lower education levels (*p* **<** 0.001**), and non-medical occupations (*p*=0.01**)) (Table 5)

**Table 5:** Association between the Davidson Trauma Scale and demographic and clinical characteristics of the studied patients

Demographic and clinical characteristics	Positive DTS		Negative DTS		***P***
	No.	%	No.	%	
Age:					
˂ 40 years old (*n* = 44)	35	79.6	9	20.4	0.7
≥ 40 years old (*n* = 45)	34	75.6	10	24.4	
Sex:					
Male (*n* = 61)	52	85.3	9	14.7	0.01**
Female (*n* = 28)	17	60.7	11	39.3	
Marital status:					
Married (*n* = 59)	49	83.1	10	16.9	0.08
Single/divorced/widow (*n* = 30)	20	66.7	6	33.3	
Education level:					
Primary, preparatory, and secondary education (*n* = 17)	8	47.1	9	52.9	<0.001**
High- and post-graduate education (*n* = 72)	61	84.7	11	15.3	
Occupation:					
Medical (*n* = 35)	32	91.4	3	8.6	0.01**
Non-medical (*n* = 54)	37	68.5	17	31.5	
Residence:					
Urban (*n* = 45)	35	77.8	10	22.2	0.9
Rural (*n* = 44)	34	77.3	10	22.7	
Chronic disease (*n* = 35)	24	68.6	11	31.4	0.1
Smoking (*n* = 27)	20	74.1	7	25.9	0.6
History of psychiatric disorder (*n* = 16)	11	68.8	5	31.2	0.4
Family history of psychiatric disorder (*n* = 27)	19	70.4	8	29.6	0.3

Test of significance: Pearson’s chi-square test

**Statistical significance

### Results of the binary logistic regression analysis of HADS scores and DTS scores with significantly related independent factors

The binary logistic regression analysis of HADS and DTS with significantly related independent factors revealed a significant model for predicting HADS and DTS scores (Table 6).

**Table 6:** Binary logistic regression analysis of HADS scores and DTS scores with significantly related independent factors

Variables	S.E.	Wald	Sig.	Odds ratio (95% CI)
**HADS–Anxiety (abnormal scores):**				
• Male sex	0.81	0.08	0.6	0.97 (0.71–2.2)
• Primary, preparatory, and secondary education (RF)	0.62	6.4	0.01**	4.5 (1.9–19.2)
• Medical occupation	0.66	2.6	0.1	0.27 (0.11–1.9)
• Family history of psychiatric disorder (RF)	0.61	5.0	0.02**	2.2 (1.07–17.3)
**HADS–Depression (abnormal scores):**				
• Male sex	0.61	1.8	0.7	0.67 (0.45–1.8)
• Medical occupation (PF)	0.52	7.4	0.03**	0.24 (0.11–0.74)
• Family history of psychiatric disorder	0.64	2.4	0.1	0.47 (0.12–2.1)
• Home isolation (RF)	0.51	7.8	0.02**	1.5 (1.1–20.9)
**DTS (positive):**				
• Male sex	0.54	1.0	0.1	1.2 (0.64–6.2)
• Primary, preparatory, and secondary education (PF)	0.64	6.7	0.01**	0.13 (0.04–0.69)
• Medical occupation (RF)	0.76	5.4	0.02**	5.2 (1.9–27.8)
• Home isolation (PF)	0.66	6.7	0.01**	0.17 (0.08–0.84)

*RF* risk factors (odds ratio > 1)

*PF* protective factors (odds ratio < 1)

**Statistical significance

Lower education levels (primary, preparatory, and secondary) and family history of psychiatric disorder were found to be risk factors for having abnormal HADS–Anxiety scores in COVID-19 patients [OR 95% CI 4.5 (1.9–19.2) and 2.2 (1.07–17.3), respectively] (Table 6).

The medical occupation was a protective factor against having abnormal HADS–Depression scores in COVID-19 patients [OR 95% 0.24 (0.11–0.74)], while home isolation was a risk factor [OR 95% 1.5 (1.1–20.9)] (Table 6).

On the contrary, the medical occupation was a risk factor for scoring positive in DTS in COVID-19 patients [OR 95% 5.2 (1.9–27.8)] while low levels of education (primary, preparatory, and secondary) and home isolation were protective factors [OR 95% CI 0.13 (0.04–0.69) and 0.17 (0.08–0.84), respectively] (Table 6).

## Discussion

A worldwide public health crisis was announced on 30 January 2020, by the World Health Organization, which then formally recognized the SARS-CoV-2 epidemic as a pandemic [29].. On 19 June 2020, the WHO documented more than 80 million SARS-CoV-2 patients from impacted nations [30]. Scientists tend to link COVID-19 to various psychiatric problems in many populations, including the infected people and the physicians who treat them [31, 32]. This would be daunting for doctors; consequently, psychological and social services are essential to alleviate physical and mental health issues in the medical community [33, 34].

Few papers investigate the psychological impact of COVID-19 on infected patients; nonetheless, none of them has demonstrated the effect of isolation on these patients. Our study is the first in Egypt to determine the psychiatric problems in home-isolated and hospital-isolated patients suffering from COVID-19 and compare them. The current research showed that most patients had initial responses of anxiety, fear, and sadness with a more negative attitude about their prognosis, with most believing that their illness will affect their future. Negative emotional responses to the disease can be illustrated by the prevalent reported data on the disease and the rapidly increasing number of deaths caused by it. Some patients [19] experienced thoughts of suicide after being infected, which was clinically more prevalent in patients in home isolation than hospital isolation (six patients).

Due to challenges caused by the pandemic, such as economic hardship, social alienation, decreased access to public medical and mental health services, and the stigma caused by infection with COVID-19, suicide rates could be elevated among home-isolated patients [35, 36].

Our results reported a statistically significant increase in the abnormal scores of HADS–Depression in the home-isolated group (69.7%) compared to the hospital-isolated group (32.6%). Moreover, we detected that 32.6% from group A and 39.1% from group B had abnormal scores of HADS–Anxiety, which means that the patients had considerable symptoms of anxiety or depression. We found 66.7% and 87.2% scored positive by the Davidson Trauma Scale (DTS) in the home-isolated group and group B, respectively, indicating the presence of post-traumatic stress symptoms.

Zhang, J and his colleagues documented a study-sized sample of 144 cases, in which they found severe anxiety (34%) and depression (28%) for patients admitted to isolation wards. Whereas other research included 26 patients, it found higher anxiety and depressive symptoms in hospital admitted patients. The third study that recruited 57 patients with COVID-19 observed that depression in recently cured (COVID-19) patients were 30% [37]. A significant sample size study (*n* = 714) of hospitalized COVID-19 patients found post-traumatic stress symptoms in 96.2% of them [6].

In a recently released meta-analysis and systematic review that incorporated 1963 studies and 87 preprints, the number of coronavirus cases was about 3559 from different countries. In contrast, there were 47 studies of SARS-CoV involving 2068 subjects, 13 studies involving MERS-CoV, and 12 reviews documenting SARS-CoV-2 (976 cases). During acute illness, the most frequent symptoms of patients diagnosed with SARS or MERS were confusion (27.9%), depression (32.6%), anxiety (35.7%), and poor memory (34.1%). By comparing the data obtained on COVID-19 patients, there was evidence of dementia (confusion 65%). After discharge from the hospital, 33% of assessed COVID-19 met the requirements for the dysexecutive syndrome [38].

Pathology of different symptoms can be distinct due to inflammation, as some studies indicate that the central nervous system may be affected by COVID-19, increasing the inflammatory immune response. In subjects with COVID-19, there is an elevation in serum C-reactive protein and high levels of pro-inflammatory cytokines and decreased total blood lymphocyte counts [38]. Neurotropic SARS-CoV-2 infection hypoxia, cerebrovascular events, and steroid therapy have impact on neurological status. These various biological mechanisms have been proposed to function as mediators of psychological impairment in COVID-19; however, there is insufficient evidence [39]. Similarly, like other physical disorders, social influences can directly intensify the psychiatric effects of exposure to COVID-19. Moreover, quarantine procedures can contribute to insomnia and psychological distress [40].

The current findings regarding the impact of the isolation setting type on presenting the patients’ psychiatric symptoms can be illustrated from various perspectives. First, hospitalized patients may have elevated post-traumatic stress levels related to the transition to a new environment. Since the human and physical hospital setting is often psychologically unhealthy, it can be loud, sensory-deprived, and disorienting. These environmental factors inhibit mobility, intensify disorientation, disturb sleep, and lead to social isolation, in addition to anxiety and apprehension. Furthermore, the deterioration of the physical condition, along with the inability to communicate with their family, fear of mortality, knowledge of the medical status of relatives and colleagues, as well as knowledge of other infected patients who died or were admitted to ICU [38, 39] can lead to increased post-traumatic symptoms in these patients. Isolation at home makes patients feel relaxed and safe with their familiar social surroundings; however, other factors may lead to adverse psychological effects, including the emergence of instantaneous stigma as a significant defect of infection due to individuals’ discrimination quarantine. This stigma may be prevalent when isolated at home and shunned by local neighbors, besides being afraid of transmitting the disease to their relatives and fear of sudden complications without receiving instant medical aid while in home isolation.

Moreover, after being discharged from the hospital, several patients treated for COVID-19 reported discrimination [40].

We found that the medical occupation has a protective effect against having abnormal HADS–Depression scores in COVID-19 patients; nevertheless, it increases the risk of having positive scores in the DTS. Some literature investigated the psychological harm caused by pandemics to medical staff who suffered from psychiatric symptoms like anxiety, depression, terror, and trauma. They reported that multiple factors, including having respiratory or digestive symptoms, negative coping style, and job burnout, participate in the anxiety or depression of healthcare workers [41]. We found that lower education levels (primary, preparatory, and secondary) were found to be risk factors for having abnormal HADS–Anxiety scores in COVID-19 patients. That was consistent with the study of Bjelland and his collaborates who found that low educational levels were significantly associated with both anxiety and depression [42].

The limitations of the current analysis must be considered when analyzing the results, which involve a limited sample size. Participants of the current research had mild to moderate cases of COVID-19 infection, in addition to the lack of appropriate reference groups to compare them with the current participants. Finally, we did not include quantitative biochemical tests, such as blood markers, to measure the inflammatory immune response.

## Conclusion

We conclude that a significant number of patients diagnosed with the COVID-19 disease develop depressive, anxiety, and post-traumatic stress symptoms, whether they were isolated in the hospital or at home; besides, the isolation setting may affect the presenting symptoms.

## Data Availability

The researchers can clarify all the data when needed.

## Abbreviations

CT: Computed tomography
SARS-CoV-2: Severe acute respiratory syndrome coronavirus 2
DTS: Davidson Trauma Scale
HADS: Hospital Anxiety and Depression Scale
MW: Mann-Whitney *U* test
PTSD: Post-traumatic stress disorder
RT-PCR: Reverse transcription-polymerase chain reaction
*χ*^2^: Chi-square test
